# 4 Ds in health research—working together toward rapid precision medicine

**DOI:** 10.15252/emmm.201910917

**Published:** 2019-09-18

**Authors:** Ellen Niederberger, Michael J Parnham, Jochen Maas, Gerd Geisslinger

**Affiliations:** ^1^ Pharmazentrum frankfurt/ZAFES Institut für Klinische Pharmakologie Klinikum der Goethe‐Universität Frankfurt Frankfurt am Main Germany; ^2^ Fraunhofer Institute for Molecular Biology and Applied Ecology IME Branch for Translational Medicine & Pharmacology TMP Frankfurt am Main Germany; ^3^ Fraunhofer Cluster of Excellence Immune‐Mediated Diseases Frankfurt am Main Germany; ^4^ Sanofi‐Aventis Deutschland GmbH, Industriepark Höchst Frankfurt am Main Germany

**Keywords:** Biomarkers & Diagnostic Imaging, Metabolism

## Abstract

Patient therapy is based mainly on a combination of diagnosis, suitable monitoring or support devices and drug treatment and is usually employed for a pre‐existing disease condition. Therapy remains predominantly symptom‐based, although it is increasingly clear that individual treatment is possible and beneficial. However, reasonable precision medicine can only be realized with the coordinated use of diagnostics, devices and drugs in combination with extensive databases (4Ds), an approach that has not yet found sufficient implementation. The practical combination of 4Ds in health care is progressing, but several obstacles still hamper their extended use in precision medicine.

Up to the end of the last century, treatment of patients was based mainly on standardized therapy of all patients with similar symptoms. However, recent leaps forward in medical knowledge have shown that this “one‐size‐fits all strategy” is far from suitable for all patients. Uniform treatment provides benefit only for a fraction of patients, while others gain no relief or even suffer from adverse effects. In the meantime, it has become clear that individual disease characteristics vary and differentiated therapy is needed at all stages of the disease. This requires specific prevention, diagnosis, treatment and follow‐up of every individual patient. Thus, in future, patients will no longer need *drugs* only but individual *solutions*. This personally adapted therapy is now within reach as a result of developments in science and technology, which facilitate more focused health care.

Terms like “individualized medicine”, “personalized medicine”, “precision medicine”, and “stratified medicine” capture the efforts being made to adjust therapeutic strategies to individual needs. However, suitable individualized or precision medicine is only possible with the well‐coordinated use of modern technologies. These include access to huge databases derived from clinical practice, as well as molecular analyses, modern and sensitive diagnostics, innovative devices for diagnosis, monitoring and drug administration, together with new and specific drugs. Thus, cooperation between disciplines is needed which cover these technologies, skills, and knowledge. Each of the 4Ds (data, diagnostics, devices, drugs) is important in its own right, but their concomitant application is even more important and could advance patient care dramatically. To date, however, the combination of the 4Ds continues to provide challenges, which need to be overcome. For instance, the varying times until approval, which range from 3 to 5 years for devices and 10 to 15 years for drugs. Furthermore, the development of devices often starts after a drug has been approved and therefore causes an inevitable delay in intellectual property and marketing. This is particularly important with regard to the fact that 60‐80% of current pharmaceutical portfolios consist of biologicals, predominantly proteins and antibodies, which cannot be administered orally, must be delivered in a relatively high volume or have problematic characteristics and therefore urgently require the timely development of novel and functional devices. Practical interactions, though, do already exist between the 4Ds, as in the “closed loop system” for diagnosis and treatment of type I diabetes. A subcutaneous blood glucose sensor (diagnostic) from which the data are transferred to a wearable data analysis system is combined with a device that calculates the insulin requirement and controls an insulin pump, which injects only the required amount of insulin (drug) into the patient's skin. This artificial pancreas is very flexible and works automatically and constitutes an advanced technique for patient treatment. However, it could be further improved by implantable micro‐devices or glucose‐sensing tattoos for instance. Consequently, by managing and coordinating 4Ds in the treatment of disease, revolutionary progress could be made in patient treatment across a wide variety of indications.

In summary, progress is being made toward better combination of drugs, devices, diagnostics, and data for the individualized treatment of patients. However, closer interactions are needed to optimize therapeutic approaches and many issues remain to be addressed.

## Drugs and treatments

### Changing development paradigms

The middle of the 20^th^ century was a period in which many new drugs were developed, mainly based on observational, phenotypic data. These drugs included antibiotics and immunosuppressive agents, which are still in use today. In the 1970s, such response‐orientated drug discovery became less important and there was a trend toward rational drug development based on newly detected biological processes. Medicinal chemistry shifted from symptom alleviation to targeting of molecular mechanisms of diseases with a focus on roles of specific proteins. Drugs developed included inhibitors of angiotensin‐converting enzyme and cyclooxygenase. Since the early 1990s, pharmaceutical productivity and new approvals have declined, despite a huge number of clinical studies, which has stimulated the search for a new paradigm for drug discovery. Concomitantly, the first tailored therapies appeared, not simply focusing on standardized blockbuster treatments, but addressing smaller patient groups or even individual patients (Munos, 2009).

### Personalizing medicine

“Stratified medicine” is defined by the National Academy of Sciences (NAS) as the aspect of personalized medicine related to grouping based on disease symptoms, drug responses, and disease properties (https://acmedsci.ac.uk/policy/policy-projects/Stratified-medicine). This strategy aims to separate patients into distinct populations according to similar biomarkers, clinical features or phenotypes, and molecular sub‐populations. A further advance is the trend toward therapies, which are specifically tailored to each individual patient. This area of personalized medicine is summarized as “precision medicine”, defined as “the use of genomic, epigenomic, exposure, and other data to define individual patterns of disease, potentially leading to better individual treatment” (Nat. Acad. Sci Eng. Med. Expert Report, dels.nas.edu/Report/Toward‐Precision‐Medicine‐Building‐Knowledge/13284). Treatment is, thus, specifically adjusted to the individual characteristics of the respective patient to correct the dysregulated molecular pathways of the disease.

In particular, the identification of the human genome sequence in 2003 opened up new possibilities to identify disease‐related disturbances/variabilities in the DNA sequence of individual patients. Genome‐wide association studies (GWAS) are now common practice to identify causal variants for respective diseases. The combination of genome sequencing and patient data from electronic medical reports (EMR) facilitates detection of relationships between genome variations and clinical outcomes. This practice has been successful in some cases and has led, for example, to a treatment approach for a genomic variant of the α2A adrenergic‐receptor (α2AAR) encoding gene, a risk factor for type 2 diabetes (Tang *et al*, 2014). However, there is still a high attrition rate for new drugs after clinical studies and the number of new approvals is disappointing (Waring *et al*, 2015). This could be due to known challenges associated with genomic and GWAS approaches. On the one hand, it is often unclear whether genomic differences are causal or incidental. On the other hand, huge numbers of polymorphisms contribute to a single specific disease, and therefore, changes in one gene may barely affect disease outcome. Therefore, the druggability of targets is often unpredictable with a high rate of false‐positive results, which impedes development of novel drugs (Baell & Walters, 2014). Furthermore, monitoring of genomic risk factors is insufficient to clarify all the multifactorial mechanisms of human physiology and pathophysiology. Disease‐inducing factors include environmental and social factors, as well as developmental and physiological components, and will need to be assessed, particularly in chronic disorders. Thus, precision medicine requires both the use of genomic and epigenome information and constitutes a platform for integration of all these data.

## Diagnostics and devices

Diagnostics and devices are inseparable partners in diagnosis and therapy. For an accurate diagnosis, combination of diagnostic biomarkers and suitable monitoring devices with relevant knowledge from databases facilitates rapid initiation of relevant drug treatment. Diagnostic errors, though, are often associated with unsuitable therapy (www.ncbi.nlm.nih.gov/books/NBK338596). Thus, there is a need for more and better diagnostics and devices to facilitate synergies between the different areas of the 4 Ds.

### Diagnostics

In spite of the enormous progress in technology and diagnostic methods, many people, particularly in developing countries, cannot access diagnostic services. Consequently, an accurate diagnosis of their disease is difficult and may lead to the wrong treatment. For instance, almost 50% of diabetes patients worldwide remain undiagnosed, increasing disease complications and high costs. A number of approaches, though, are being taken to develop specific tests for rapid accurate diagnosis of acute disorders as well as prophylactic diagnosis of chronic diseases. A suitable diagnostic should be predictive, delivering accurate and reliable results already at an early stage or before the onset of the disease. At best, it should be available directly at the point of care (PoC), allowing for rapid and directed initiation of therapy or prophylaxis in acute conditions and might enhance survival rates in critically ill patients. Furthermore, it should be low cost and easily handled, usable by every physician or even the patient. Digital solutions clearly offer bigger advantages, requiring the involvement of device manufacturers.

### Biomarkers

Specific biomarkers for subgroups of patients are crucial for the development of suitable diagnostics for individualized therapy. Thus, development of novel diagnostics usually starts with identification of biomarkers, including circulating DNA, RNA, proteins, lipids, and components of the microbiome or metabolites in body fluids or disease‐relevant tissues. These should correlate with disease onset and progression and be monitored either individually or in combination. Ideally, such biomarkers are also suitable for monitoring the response to therapy. The analysis of diagnostic markers is often restricted to complex laboratory methods such as genotyping, RT–PCR, proteome analysis, mass spectrometry, immunohistochemistry, or *in situ* hybridization. Here, precise and easily usable devices could drastically speed up and facilitate diagnosis. Emerging technologies include robotic cameras for inaccessible anatomical sites, optical imaging techniques, disposable electrochemical chips, and inexpensive consumer multi‐gene analysis. Currently, drug therapy may be linked with companion diagnostics, to identify specific biomarkers to support the choice of a safe and efficacious medication. FDA‐approved companion diagnostics comprising *in situ* hybridization or immunohistochemistry are already in use and proving helpful (Dugger *et al*, 2018). Next‐generation diagnostics based on Crispr‐Cas biology have been introduced recently. With this approach, detection and differentiation of various viruses is possible. The method is relatively easy, allowing rapid and accurate determination of pathogens in body fluids. It might thereby further facilitate early diagnosis of infection and immediate or even prophylactic treatment when fully evaluated and approved for clinical use (Chertow, 2018).

### Devices

Medical devices can be defined as “instruments” for either drug delivery (therapy) or diagnosis to diagnose, cure, mitigate, treat, or prevent disease in humans. They need to be fully clinically evaluated and may be low‐risk, non‐invasive or high‐risk, invasive devices. Here, we focus mainly on devices for facilitation of diagnosis, drug administration, and drug monitoring at the PoC. An optimal device for disease/health control should be affordable and easy to handle, portable, and automatic and allow sensitive, specific, and rapid determination of the required parameters. Several such devices, such as urine sticks for determination of glucose, pH, or protein, have been in use for many years. However, more sophisticated tools for rapid diagnosis and PoC testing are continuously being developed (Table 1). Considerable progress has been achieved with simplified biosensors and several types of invasive and non‐invasive sensors for analyses of blood, saliva, sweat, exhaled breath, and eye fluid. Other possibilities include the use of microchips. A lab‐on‐a‐chip assay allows for rapid and automated identification of several different parameters, including bacterial species and detection of antibiotic resistance in minimally processed body fluids. This can be done with a DNA array on a microchip or micro‐immunoassays, which allow identification of different genes and SNPs or disease‐specific antibodies in a small volume of biofluid, respectively. These analyses also work as paper‐based analytical devices with the advantage of being relatively cheap, quickly produced, and easy to use. It is likely that their use will advance further when multiplex targets can be simultaneously determined and standard applications are routinely connected to barcode scanners or smart phone applications for barcodes. However, most of the new developments are far from the market and need further extensive testing until they are fully functional for a broad range of applications.

**Table 1:** Some emerging approaches to integrated diagnosis for rapid personalized therapy

• Highly specific, rapid diagnosis of acute disorders • Prophylactic, predictive diagnosis of non‐malignant chronic diseases • PoC devices for multiple markers (DNA, RNA, metabolites) • Non‐invasive electronic sensors (e.g., sensor for hemoglobin) • Lab‐in‐a‐drop • Simplified (fluorescence/colorimetric) biosensors • Paper‐based microfluidics (e.g., for PCR in blood, recording on a mobile phone)

### Health care devices

**Figure 1:**
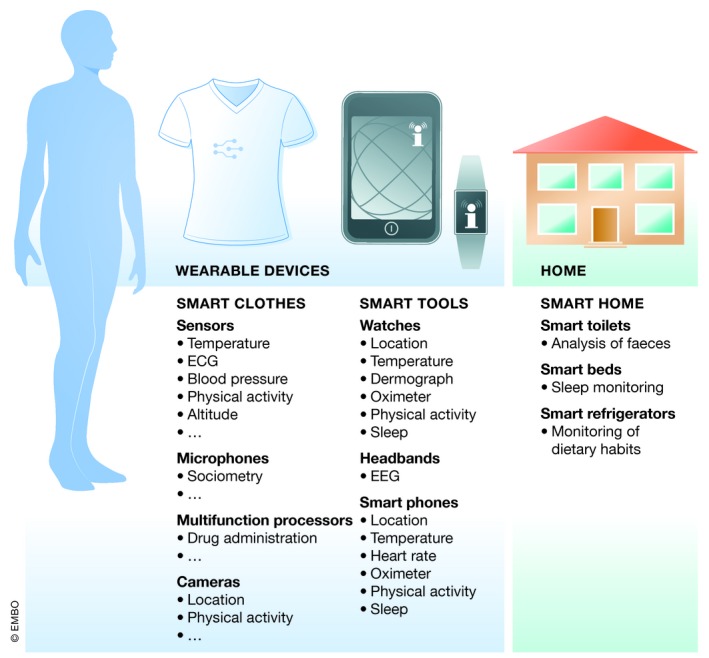
Health care devices Health monitoring by wearable devices or smart homes. Examples are given of possibilities for smart clothes and smart tools, which are mobile and deliver information from wherever a person is located. In smart homes, there are several fixtures and fittings, which could be used for passive monitoring of several health parameters.

At present, healthcare systems focus mainly on patients with clear symptoms or on diagnosis of a disease. However, treatment alone of symptoms or molecular pathways of existing disease is insufficient, since prevention and avoidance of diseases has become a major emphasis. Current developments in precision medicine, therefore, are directed toward preventive documentation of individual health risks or at least early detection of disease. Wearable, consumer‐friendly devices (for healthy people as well as patients) allow for monitoring of human physiology and pathophysiology and are increasingly employed. The initiative started mainly with healthy people wanting to control their lifestyle, for example, with smart watches or fitness trackers who now track physical activity, monitor sleep, or analyze images and voice. The scenario will be further enhanced by devices in the individual′s home, which monitor physical and mental health, thereby creating a “smart home” (Fig 1) (Gambhir *et al*, 2018). Wearable devices for monitoring and diagnosis of diseases, for instance, in the cardiovascular, metabolic, or psychological sectors are now available (Guk *et al*, 2019). The diversity of the devices is relatively high, ranging from simple trackers for movement or body weight to highly sophisticated long‐term monitors to analyze vital signs over a long period of time. Apart from using wearable devices separately, they can be combined for multi‐pattern measurement and early identification of changes in health status. At a more advanced stage, these devices optimally can be coupled to drug administration systems, as in the diagnosis and treatment of diabetes, as mentioned above. At present, many devices are still under development and need extensive testing before entering clinical application. Such devices for chronic inflammatory disorders would be a big step forward.

### Drug administration and monitoring

In addition to monitoring health and disease parameters, devices are also used for drug administration and therapeutic drug monitoring (TDM). Most biologicals, including antibodies, and synthetic molecules such as peptides and oligonucleotides, cannot be administered orally and rely on devices such as injection pens, autoinjectors or pumps, nebulizers, or transdermal patches, where applicable, connected directly to a diagnostic tool.

Therapeutic drug monitoring (TDM) supports the control of efficacy and toxicity, as well as adjustment of drug dose. This is crucial for a number of drugs such as antiepileptics, antibiotics, or immunosuppressants, with unpredictable pharmacokinetics and narrow therapeutic ranges. To date, TDM is based mostly on immunoassays and chromatography. However, like most other diagnostics, laboratory equipment and expert personnel are essential. Cheap and easy to use devices for quantitation of drug levels in the blood are currently not commercially available, although they would undoubtedly improve safety and efficacy of administered drugs and allow for dosage adjustment at the bedside or in the patient's home. Although there are several promising developments, there are few sensitive and reliable biosensors. Those available detect major metabolites and electrolytes in the blood, but detection in “uncommon” body fluids, such as sweat or saliva, still requires prior preparation to concentrate the respective biomarkers. Furthermore, hormones, peptides, etc., as in endocrine or inflammatory disorders, are currently undetectable at all by PoC. Therefore, most sensors still require further optimization and assessment in human trials before being approved and used routinely.

## Data

In the dawning era of precision medicine, collaborations between scientists, clinicians, and patients will generate multidimensional data sets from huge cohorts of patients, healthy individuals, and experimental organisms. These data must be analyzed and integrated into networks and databases to be made accessible to specialized interest groups. The expected outcome is a form of atlas, already described as “Google map for health” by the US National Academy of Sciences (NAS). This health map must be a dynamic and flexible tool to connect molecular research and clinical data but will then facilitate the combination of risk assessment and customized monitoring of health or disease to enhance prevention strategies, therapy, and health services for each patient.

The term “Big Data” encompasses a large volume of high diversity data from single individuals to large cohorts on biological, clinical, environmental, and lifestyle information. Inevitably, this raises the challenge of how to handle and interrogate such megadata. Following capture, data must be curated—stored, analyzed, presented, and coordinated. The information must be interpreted and then translated into diagnostics and therapeutic action. This demands the introduction of support systems and training programs for clinicians to translate data into clinical decisions and workflows, thus facilitating therapy selection to fit the patient's needs precisely.

**Figure 2:**
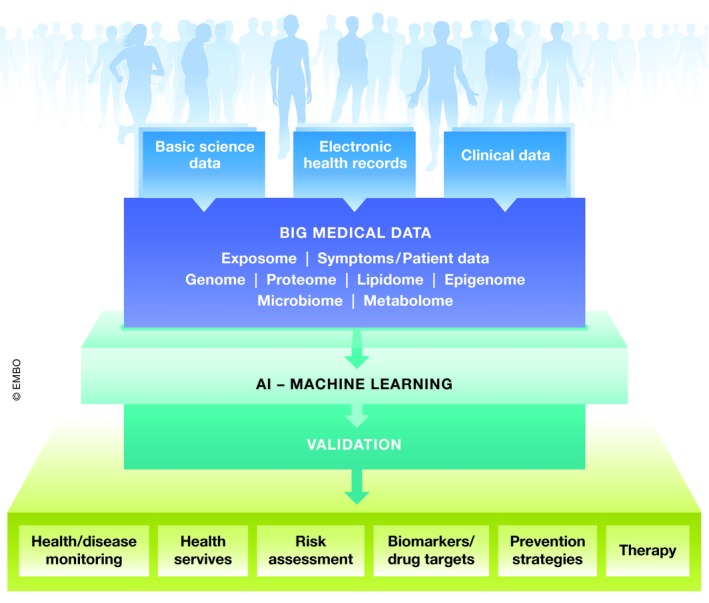
Big Data as a hub between data acquisition and therapeutic outcome

*Machine learning* (deep learning) represents the virtual branch of artificial intelligence (AI) and relates to the ability of a computer to learn from data/experience without human input. This is particularly important in the healthcare system where AI is able to provide continuous process improvements by generating information as well as health management systems. This is done, for instance, by integrating and analyzing electronic health reports (EHR), which provide information on the patient's family history of hereditary diseases or increased risk to develop a chronic disease. Subsequently, in terms of precision medicine, the system is able to support diagnosis of diseases and the physician's therapy decision. In some aspects, AI works faster, with greater consistency and higher reproducibility than humans (He *et al*, 2019), and might, therefore, provide synergistic benefit for patient treatment in combination with clinicians (Fig 2).

Furthermore, the vast quantities of *genomic data* can be used for computational development of novel therapies. In particular, genome‐wide association studies (GWAS) are powerful tools to detect genomic variants as inducers of diseases. However, GWAS require large sample numbers, are largely unpredictable, and necessitate many follow‐up studies to confirm their impact on a disease. Consequently, for precision medicine, the data available to medicine should include multi‐omics profiling from an individual's genetic makeup (genome), combined with factors such as gene expression (transcriptome), protein identification (proteome), metabolism (metabolome), and gut microflora (microbiome). Increasingly, omic analyses now comprise investigation of the methylome, acetylome, lipidome, toponome, proteome, etc., combined with deep phenotyping of patients. Moreover, it is very important to integrate changes in epigenomic data, induced by lifestyle or environmental conditions.

*Patient‐reported data* from clinical treatments, medical practices, patient registries, patient forums, and social networks should be collected. These individual data can be added to data networks to complement data on health or disease status, requiring the collaboration of patients and healthy individuals who may have privacy issues with sharing their personal data. Clinical data can be shared anonymously in electronic health registries (EHRs), providing a longitudinal profile of patient outcomes and medication, comorbidities, etc. It has been calculated that EHRs can drastically reduce costs, shorten study time, and deliver valuable patient data. Big Data may also increase the predictive power of biomarkers by using information from the literature and databases, combining them and then creating multidimensional signatures. An optimal network would provide insight both into incoming data and curated output, allowing adaptation of the system. These “dynamic knowledge repositories” would contribute to stratification of patients, detection of patient relationships, and the integration of these data into clinical practice. In combination with AI informatics tools, mistakes would be reduced and patient therapy adapted accurately.

## Connecting 4Ds in precision medicine

As indicated above, patient treatment strategies are changing radically toward individualized therapy. This rethinking is accompanied by significant technical progress in digitalized laboratory methods, in patient‐ and physician‐friendly diagnostics and devices and most impressively, in data acquisition and analysis. Today, with sophisticated software, extensive data generated from a single experiment or study can be integrated into databases and data from preclinical and clinical studies interrelated with case reports and then incorporated into diagnosis of diseases and treatment decisions. Thus, adapted therapy of patients can now be based on their lifestyle, genome, and specific individual disease mechanisms. However, a variety of hurdles remains to be overcome. First of all, the huge volume of data is complex to handle, access, store, and secure; standardization of data is limited; and different data sets are often difficult to compare. Due to ethical and legal restrictions, access to patient data is restricted and cannot be accessed for the development of diagnostics, devices, and novel drugs. Only after data are officially approved and released can these components be implemented, thus delaying their use for clinical development. In addition, very sophisticated approaches must be taken to develop new diagnostics and devices, but most of these novel tools are still in their infancy. Devices for diagnosis of blood glucose in diabetes have advanced to the point where PoC is already a thing of the past and wearable peripheral devices are commonplace. In diabetes, a future goal is the development of non‐invasive biosensors. However, for most other diagnostic biomarker analytes, no wearables are available at all (Heikenfeld *et al*, 2019).

**Figure 3:**
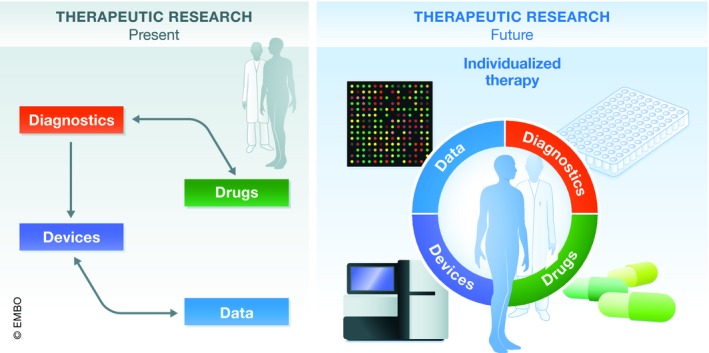
Patient therapy at present and in a future perspective Scheme of development of therapies at present and with a future perspective, indicating improvements in interactions between the 4Ds to facilitate individualized therapy.

The integration of engineers and specialists in informatics into life sciences has already led to strong improvements in health care, in development of devices as well as for imaging methods or genome sequencing. Nevertheless, these interactions need to be intensified to support further interconnections between medical and engineering education. This could lead to new inventions for precise and high‐quality medicine: on the one hand, improving diagnosis, treatment, or even prevention of diseases; and on the other hand, reducing costs in the health system. Generally, it is clear that better interactions and coordination between the 4Ds could lead to dramatic progress in the generation of improved drug therapy in the coming years (Fig 3).

## Discussion

Novel data processing, research, and engineering technologies have opened up new opportunities to improve health care and develop individualized therapies. This precision medicine allows treatments that consider a patient's genetic, anatomical, and physiological makeup. Genome sequencing or genotyping and advanced diagnostic abilities, with the support of modern devices, facilitate faster diagnosis and early initiation of efficient therapy, often directly at the bedside. The generation of “Big Data”, using modern “omics” technologies as well as the extensive collection of patient data, permits huge databases to be built as a basis for further progress in the development of drugs, diagnostics, and devices.

However, it is clear that all these new developments come with severe challenges. Firstly, it is unclear how many data sets need to be combined for better diagnosis and therapy, particularly for complex chronic disorders. Too few data provide no advantage, but information overload might complicate the situation. Continuously accumulating data must be stored securely and be available simultaneously for the therapy decision. A further challenge with Big Data is their statistical complexity and the assessment of their significance. Even highly specialized physicians have neither the time nor training to directly correlate concurrent data for a therapy decision. Therefore, development of software‐based support for PoC decision‐making is crucial for the success of precision medicine. AI might provide such possibilities in the future, though currently, clinical use of AI is hampered by its complexity and issues concerning data sharing, transparency, and patients safety (He *et al*, 2019; Topol, 2019). Nevertheless, the IDx‐DR software program has been approved by the FDA as the first autonomous diagnostic system for screening of diabetic retinopathy without clinical interpretation (He *et al*, 2019).

Unfortunately, at present, datasets tend to be biased toward accessible organ and cell systems and both the quality of publicly available data and the reproducibility of experiments vary, making interpretation difficult. Moreover, all computational predictions must be validated in experimental and clinical settings. This could be solved by increasing standardization of methods with GLP or GLP‐like protocols. Here, collaboration between clinicians, clinical biochemists, bioinformaticians, and quality control experts would be most productive. A further problem is that most samples are taken from patients with previously clinically diagnosed pathologies, so that basal levels of biomarkers in healthy subjects are unknown. For this, research funding agencies are called upon to take action.

To gain a broad overview of individual health status, data from all aspects of the life of an individual must be collected and combined over the whole lifetime. To date, apart from several long‐term clinical cardiovascular studies, findings are limited and most of the data on diet or environmental factors, for instance, are not taken into account. In future, this continuous monitoring of a set of parameters in daily life might be possible with wearable devices or by a “smart home” (Gambhir *et al*, 2018). A bias in the medical systems, for instance in prediction of disease outcome or treatment strategies, exists since minor populations are often overlooked in data acquisition. This will be improved with more accessible data from as many individuals as possible (Price & Cohen, 2019).

In addition, there are several ethical problems with data collection. Use of data from individual health monitoring and diagnosis requires patient consent and can raise problems with privacy protection. Property, intimacy, and equity must be preserved and discrimination, and the creation of stigma strictly avoided. Thus, public support, which is extremely important for collection of data, can only be achieved through clarity of data control and appropriate and clearly formulated procedures with controlled standards for data mining, use, and safety. Moreover, the mode of patient consent has to be clearly defined. Patient consent for every set of data generated for each specific purpose would drastically increase administration problems, while non‐consented use, mainly of unidentifiable data, might bring benefit to the patients, but again constitutes a form of privacy violation when not clearly regulated (Price & Cohen, 2019).

Currently, data collection and analyses are expensive tools and will raise the costs of new therapies. This could lead to improved health accessories but only for a small proportion of privileged persons. Fortunately, efforts are being made to reduce the costs of research and analyses and high hopes are pinned on engineers to make this possible. Obviously, education of all personnel involved in the development process is absolutely essential for the progress of individual therapies. In future, medical engineers will need to understand both clinical and technical aspects, combining this knowledge to facilitate transformation to data‐driven, mechanism‐based health and health care for each individual.

**Box 1: Definitions**

*Drug*: a chemical or biological product used for the diagnosis, cure, mitigation, treatment, and prevention of diseases.

*Device*: Instrument used either for drug delivery or diagnosis, which contributes to cure, mitigation, treatment, and prevention. Devices are characterized by the absence of chemical action and are not metabolized to chemically active compounds.

*Diagnostics*: Biosensors or bioinformatics systems, which might include portable devices (FDA description: *in vitro* diagnostic that provides information that is essential for the safe and efficacious use of the corresponding therapeutic product).

*Data*: digitalized information contributing to the development of devices, drugs, diagnostics, and artificial intelligence.

## Conflict of interest

The authors declare that they have no conflict of interest. JM is an employee of Sanofi.
